# Community‐engaged crime prevention through environmental design and reductions in violent and firearm crime

**DOI:** 10.1002/ajcp.12802

**Published:** 2025-03-18

**Authors:** Laney A. Rupp, Shaun Bhatia, Daniel B. Lee, Rachel Wyatt, Gregory Bushman, Thomas A. Wyatt, Jesenia M. Pizarro, Caroline Wixom, Marc A. Zimmerman, Thomas M. Reischl

**Affiliations:** ^1^ Department of Health Behavior and Health Equity, School of Public Health University of Michigan Ann Arbor Michigan USA; ^2^ Institute for Firearm Injury Prevention, University of Michigan Ann Arbor Michigan USA; ^3^ Neighborhood Engagement Hub Flint Michigan USA; ^4^ School of Criminology and Criminal Justice Arizona State University Phoenix Arizona USA

**Keywords:** busy streets theory, community coalition, community engagement, CPTED, firearm violence prevention, program evaluation, violence prevention

## Abstract

In the U.S., crime and violence are concentrated in cities that have lost industry and population due to economic disinvestment and structurally racist policies. Researchers, practitioners, and policymakers have called for community‐level approaches that reduce violence in these cities by improving unsafe physical environments, increasing social equity and cohesion, and engaging community members in neighborhood change. We tested Busy Streets Theory by examining how community‐engaged Crime Prevention through Environmental Design (CPTED) strategies implemented by a community coalition may reduce violent and violent firearm crime incidents in Flint, Michigan, a legacy city in the Midwestern U.S. We used linear mixed effects regression models to examine how the annual aggregate intensity of physical and social CPTED activities from 2015 to 2018 was associated with changes in annual violent crime levels from 2016 to 2019 for 505 street segments in Flint, MI. After adjusting for baseline violent crime density, neighborhood disadvantage, property maintenance, and spatially lagged violent crime density, we observed that higher levels of community‐engaged CPTED intensity were associated with steeper declines in violent crime density over time (*β* = −0.14, *p* < 0.001). Similarly, higher levels of community‐engaged CPTED intensity were associated with steeper declines in violent firearm crime density over time (*β* = −0.19, *p* < 0.001). The results suggest the vital role that creating busy streets through community‐engaged CPTED may play in community violence prevention.

## Highlights

- Greater intensity of community‐engaged CPTED activity is associated with decreasing violent crime
- Greater intensity of community‐engaged CPTED activity is associated with decreasing firearm crime
- Community‐engaged CPTED is a promising intervention to prevent violence in disinvested neighborhoods

Violent crime poses an urgent public health challenge in the United States with long‐term consequences for the health and well‐being of communities (American Public Health Association, 2018; Bauchner et al., 2017). Exposure to violence disrupts healthy youth development (Gabarino et al., 2002) and is associated with adverse mental health symptoms (Gill, 2002; Ranney et al., 2019), substance use (Walton et al., 2017), and elevated risks of cancer and other chronic diseases (Hsieh et al., 2017; Wilson et al., 2004). Violence exposure is among the most robust predictors of future involvement in violence, including increased risks of assault injuries and criminal justice involvement (Carter et al., 2015; Cunningham et al., 2018; Gabarino et al., 2002; Rowhani‐Rahbar et al., 2015; Schmidt et al., 2019). Firearm injuries became the leading cause of death for children and adolescents in the U.S. in 2020. Firearm homicides, in particular, rose by more than 33% from 2019 to 2020 (Goldstick et al., 2022; WISQARS, 2019). Consequently, interventions addressing violence and firearm violence are urgently needed (FBI National Press Office, 2021; Goldstick et al., 2022).

In the United States, violence and firearm violence are concentrated in legacy cities that have lost significant industry and population and where historical and ongoing structural racism contribute to economic disinvestment and health disparities (Cunningham et al., 2018; Jacoby et al., 2018; Knopov et al., 2019; Mehranbod et al., 2022; Uzzi et al., 2023). Disinvested cities with concentrated property vacancy are particularly vulnerable to violent crime because illegal activity can occur in depopulated areas with limited surveillance, and weapons like firearms can be concealed in vacant lots or buildings (Branas et al., 2013; Garvin et al., 2013; Spelman, 1993). Physical disorder in these settings signals a lack of social control, which increases fear of crime and undermines protective social ties (Bursik & Grasmick, 1999; Garvin et al., 2012; Perkins & Taylor, 1996; Ross & Mirowsky, 1999; Taylor et al., 1985). Criminal justice interventions such as surveillance, arrest, and incarceration are common responses to violence in these places; however, they may result in negative consequences such as the breakdown of social control in communities (Rose & Clear, 1998). To address place‐based risks for violence, researchers, practitioners, and policymakers have called for nonpunitive, community‐level approaches that secure unsafe environments, increase equity and cohesion, and engage community members in intervention development and delivery (American Public Health Association, 2018; Cunningham et al., 2019; Hibdon et al., 2021; Ngo et al., 2019; Pizarro et al., 2020).

Interventions that remediate and improve vacant and blighted properties, including mowing and landscaping vacant lots, repairing the façade of abandoned buildings, and demolishing unsafe structures, are a promising alternative to punitive criminal justice strategies. These interventions have shown promise for reducing fear of crime (Branas et al., 2018; Burt et al., 2022), violent crime (Branas et al., 2018; Heinze et al., 2018; Kondo et al., 2016; Locke et al., 2023; Pizarro et al., 2020), and firearm violence (Branas et al., 2016; Bushman et al., 2023; Jay et al., 2019). A growing number of researchers have also examined the crime prevention effects of community‐engaged interventions to improve neighborhood built environments. Heinze et al. (2018) found that street segments receiving consistent, community‐engaged mowing of vacant lots reported a 40% reduction in assaults relative to street segments with no maintenance. Gong et al. (2023) found steeper reductions in crimes surrounding vacant lots that were repurposed by residents (e.g., converted into community gardens or playfields) than near lots that were landscaped by professional contractors. Bushman et al. (2023) found that vacant properties mowed by community groups experienced reductions in firearm‐involved crime over time, while vacant properties mowed by professional crews did not.

These findings align with Busy Streets Theory, which posits that residential collaboration in neighborhood improvement not only creates safer physical contexts, but also catalyzes social processes that can attenuate violent and firearm‐related crime (Aiyer et al., 2015). In prior studies, we have found that community‐engaged neighborhood improvement efforts are positively associated with the development of social resources helpful for deterring crime, such as an increased sense of community, social capital, and collective efficacy (Alaimo et al., 2010; Rupp et al., 2020). For example, residents in Flint, MI who collaboratively planned and implemented neighborhood improvements (e.g., boarding abandoned homes, mowing vacant lots) reported feeling closer to neighbors and relying on each other to solve problems (e.g., sharing tools) (Rupp et al., 2020).

## Community‐engaged crime prevention through environmental design

In the current study, we examined how engaging community members in a promising neighborhood‐level strategy, Crime Prevention Through Environmental Design (CPTED) (Cozens & Love, 2015), was associated with violent crime trends. CPTED is an interdisciplinary intervention—drawing from urban planning, criminal justice, and public health— that improves physical conditions in neighborhoods to deter crime and violence. Early CPTED strategies focused on natural surveillance, which included improving sightlines to make activity more observable (Jacobs, 1961) and increasing territoriality by modifying spaces (e.g., installing fences, landscaping) to signal ownership and increase perceived risks of offending (Jeffrey, 1971; Newman 1972, 1996). While considerable overlap exists between other built environment interventions (e.g., vacant property greening) and CPTED, CPTED is differentiated by including a systematic assessment of the built environment and applying a suite of physical strategies to create “defensible space” (Newman, 1972). Creating defensible space entails cueing residents to care about an area and creating or enhancing opportunities for residents to monitor and protect it (Cozens & Love, 2015; Newman, 1996). These strategies are based on the theoretical assumption that individuals are rational actors who weigh the consequences of their actions and are dissuaded from criminality when the potential punitive consequences outweigh the potential rewards (Clarke, 1989; Jeffery & Zahm, 1993). A recent review of CPTED studies and concepts, however, noted criticisms of interventions that were physically deterministic (physical CPTED) and emphasized the need to also change social environments (social CPTED) (Cozens & Love, 2015; Saville & Cleveland, 1997). This review presented a conceptual model integrating physical and social CPTED.

The conceptual model developed by Cozens and Love (Cozens & Love, 2015) highlights several defining characteristics of physical and social CPTED. Regarding physical CPTED, six core strategies are proposed: *Territorial Reinforcement* strategies use elements such as signage, decorative fencing, and landscaping to convey responsible ownership, delineate between public and private property, and create defensible space*. Maintenance* strategies apply routine care and upkeep of physical spaces to increase positive perceptions, signal social control, and reduce crime‐attracting disorder (e.g., removing graffiti, mowing overgrown lots). *Surveillance* strategies include clearing sightlines (e.g., removing overgrowth) and adding lighting or patrols to make the activity more observable and increase the perceived risks of offending. *Access Control* strategies direct movement through public spaces using elements such as paths and archways to indicate preferred access or to limit unwanted access with barriers (e.g., boarded windows). *Target Hardening* strategies refer to more involved elements that block access to crime targets, such as fences, security doors, locks, and guards. In this study, we consider target hardening to be an intense form of Access Control, due to the considerable conceptual overlap between these strategies (Cozens & Love, 2015). Finally, *Legitimate Activity Support* strategies apply design elements that communicate norms for appropriate use of spaces (e.g., trash cans, directive signage), encourage positive uses (e.g., playground builds), and invite the presence of positive users to observe activity and deter crime.

As described by Saville and Cleveland (2013), social CPTED strategies involve leveraging support from more resourced organizations to increase social cohesion, collective efficacy, and behavioral action, thereby fostering neighborhood empowerment (Aiyer et al., 2015; Reynald, 2011; Saville & Cleveland, 2013). Saville and Cleveland (2013) originally proposed four social CPTED strategies: social cohesion (which includes two components of positive esteem and social glue) community culture, connectivity, and community threshold capacity. In this study, we include three of the four strategies and have adapted their names to better reflect how we operationalized them. *Social Cohesion strategies*, formerly ‘positive esteem,’ enhance connectedness by engaging residents in relationship‐building activities (e.g., conflict mediation, neighborhood meetings) that nurture social ties, inclusiveness, respect, and collaboration. *Resident Involvement* strategies, formerly ‘social glue,’ engage residents in attending, planning, and implementing CPTED strategies. *Community Events*, formerly ‘community culture,’ activate places with programs and activities (e.g., festivals, celebrations) that highlight local traditions and values to build shared identities and a sense of community. *External Support* strategies, formerly ‘connectivity,’ mobilize resources (e.g., funding, in‐kind support) and partnerships with local organizations and institutions (e.g., hospitals, police) to improve physical and social environments. *Community Threshold Capacity* strategies work to balance land use to increase community safety and limit crime attractors (e.g., liquor stores). We elected not to include Community Threshold Capacity in our study, as it was not as relevant to the interventions we evaluated. Finally, we included *Capacity Building* as a new social CPTED strategy not originally conceptualized by Saville and Cleveland (2013). This strategy involves training residents and community partners to systematically assess community sites such as residences, businesses, and places of worship and apply CPTED strategies to improve their safety.

## Prior CPTED evaluations

Evaluations of CPTED strategies have demonstrated positive effects for crime reduction (Gardiner, 1978; Newman, 1996; Poyner, 1983), including reductions in burglary, robbery, and injuries resulting from robberies (Casteel & Peek‐Asa, 2000; Farrington et al., 2002; Hedayati Kushmuk & Whittemore, 1981; Marzbali et al., 2016; Murphy & Eder, 2010; Poyner, 1983). Early researchers in this field mainly employed case study methodologies. For example, Poyner (1994) found that modifying landscaping and housing layout to increase territoriality helped to prevent residential burglaries, robberies, and purse snatchings. Painter (1994) found that adding street lighting to enhance surveillance was associated with a decrease in burglaries and property crimes. Matthews (1990) found that access control measures such as road closures were associated with decreased open‐air prostitution. Yet collectively, CPTED evaluation studies are limited by methodological constraints, including small samples (Casteel & Peek‐Asa, 2000), short follow‐up periods (Gardiner, 1978), and reliance on self‐reported victimization data (Vagi et al., 2018). Most studies used pre‐post survey designs that specified commercial buildings or residences as the unit of analysis but did not capture the crime prevention effects of CPTED interventions in surrounding areas (Casteel & Peek‐Asa, 2000). Moreover, CPTED evaluations have not adequately controlled for neighborhood‐level correlates of crime, and researchers rarely addressed the spatial nature of the data, for example, by testing and accounting for spatial autocorrelation among crime incidents.

Rigorous evaluation of CPTED has been further limited by inadequate measurement (Ekblom, 2011; Lee et al., 2023). Observational tools have been applied to catalog the presence of CPTED‐related features at residences, schools, and streets (e.g., presence of lighting, aesthetic conditions of buildings) (Hedayati Marzbali et al., 2016; Lee et al., 2023), but have not been used to evaluate the effects of CPTED interventions implemented to prevent crime in neighborhoods. Additionally, while CPTED projects vary widely in terms of the type and number of strategies applied, few researchers have evaluated the effects of all CPTED strategies applied together (Abdullah et al., 2013; Lee et al., 2023), or assessed the intensity of their application. In a review of CPTED evaluation studies, Casteel and Peek‐Asa (2000) found that interventions that used more than one CPTED principle were associated with greater reductions in robbery than interventions that applied only a single strategy. Similarly, Vagi et al. (2018) found that students at public middle schools with more in‐depth CPTED interventions reported higher perceptions of safety and lower levels of violence perpetration relative to students at schools with less in‐depth interventions. Yet to our knowledge, no measure exists to score the intensity of neighborhood‐level CPTED interventions incorporating both social and physical CPTED.

Finally, few researchers have examined the effects of using CPTED strategies to reduce violent and violent firearm‐related crime at the community‐level (i.e., within larger geographic areas like neighborhoods rather than single commercial, residential, or school sites). Carter et al. (2003) found that combining CPTED strategies with increased police patrols reduced calls for prostitution and police service in a high crime corridor but found no significant reductions in violent crimes. Lee et al. (2023) inventoried neighborhood features related to CPTED strategies on street segments surrounding economically vulnerable schools. In this cross‐sectional study, they found that maintenance features, including the overall cleanliness of streets and the visual quality of buildings, were negatively associated with violent crime. Given the limited nature of the extant research, our study sought to address several methodological and conceptual gaps.

## Present study

We examined community‐engaged CPTED approaches that applied both physical and social CPTED strategies. We examined whether different levels of community‐engaged CPTED activity intensity (i.e., the aggregate of physical and social CPTED intensity) were associated with trends in violent and firearm crime density on street segments in Flint, Michigan, a postindustrial legacy city with high concentrations of vacant property. As the CPTED intervention was implemented by a community coalition and guided by community‐driven priorities, we noted considerable variation in the intensity of CPTED activities implemented across street segments. This natural variation provided an opportunity to evaluate the effects of different levels of CPTED intensity on violence.

Street segments, defined as both sides of a street between two intersections or between an intersection and an end of the street, are a useful spatial unit of analyses for studies of crime prevention in cities (Weisburd et al. 2012, 2016). Taylor (1997) argued that street segments operate as influential behavior settings where residents know each other, and are aware of shared norms, and behavior patterns. Street segments can also differ in property maintenance, social interactions, and collective efficacy (Sampson et al., 1997; Weisburd et al., 2020). These differences are relevant to place‐based crime risks specified in rational choice theory (Clarke & Felson, 2011), which asserts that offenders seek out physical and social environments that provide the most favorable opportunities to commit crimes without being detected (Wilcox & Cullen, 2018). We hypothesized that street segments in our study area with more intensive CPTED activities would have steeper decreases in violent and violent firearm crime over time than street segments with less intensive CPTED activities.

## METHODS

We used linear mixed effects regression models to examine how the annual aggregate intensity of physical and social CPTED activities from 2015 to 2018 was associated with changes in annual violent crime levels between 2016 and 2019 for 505 street segments in Flint, MI. The regression models also controlled for contextual factors related to violent crime including: (A) averaged baseline violent crime in 2014 and 2015, (B) a neighborhood disadvantage index based on US Census data (Burt et al., 2022; Sokol et al., 2022), (C) building and lawn maintenance ratings for every parcel on each street segment (Reischl et al., 2016), and (D) spatially lagged violent crime levels.

### Study setting

The study occurred in a central area of Flint referred to as the University Avenue Corridor (UAC). The UAC area is approximately two square miles stretching west from downtown Flint and anchored by key institutions including Kettering University, the University of Michigan‐Flint, Hurley Medical Center, and McLaren Regional Hospital. In 2012, these anchor institutions worked with neighborhood associations, social service organizations, local law enforcement, and other community partners to create the University Avenue Corridor Coalition (UACC) with the mission to transform the area into an attractive and crime‐free community conducive to sustainable development. Over the course of this study, the UACC grew into a cross‐sector partnership with more than 100 member organizations. Notably, 1 year before the UACC formed, Michigan's governor had declared a financial state of emergency for the City of Flint due to ongoing budget deficits and an emergency manager controlled the city's finances from 2011 to 2015. During this time, the city experienced budget cuts and reduced municipal services, including law enforcement. One goal of the UACC was to support community‐driven and institution‐backed strategies to promote neighborhood health and safety in the face of reduced law enforcement services.

Early in the formation of the UACC, coalition partners embraced CPTED strategies as a key component of their crime prevention efforts. One of the institutional partners (Kettering University) led an effort to receive a Byrne Criminal Justice Innovation (BCJI) Program grant from the US Department of Justice, which provided 3 years of funding to implement CPTED strategies on individual parcels, along streets, and in specified areas (e.g., groups of city blocks, neglected parks). Additional funding from the Centers for Disease Control and Prevention and in‐kind contributions supported the implementation of CPTED activities within the UAC. For further details about the UACC's formation and CPTED implementation in the UAC, see Rupp et al. (2020).

### Measures

#### CPTED activity intensity

The UACC project manager (a certified CPTED trainer) worked closely with the research team to record 373 CPTED activities that occurred in the UAC from 2015 through 2018. Research team members periodically reviewed these activity records with the UACC project manager and recorded activity descriptions, dates, community partners engaged, resources contributed by those partners, number of volunteers engaged, and the number of community members participating. The UAC project manager also estimated total activity costs, inclusive of labor, equipment, and materials. Activity costs were estimated in ordinal ranges (i.e., low‐cost activities: <$500; medium‐cost activities: $500–$5,000; and higher‐cost activities: >$5,000). Based on these activity descriptions, and CPTED concepts reviewed by Cozens and Love (2015) and Saville and Cleveland (2013), the research team developed ordinal intensity ratings for each CPTED strategy (described below). The ordinal intensity ratings were assigned to all CPTED activities by the research team, using consensus coding procedures based on combinations of intensity dimensions relevant to each CPTED strategy. To rate the intensity of *Physical CPTED* activities, the research team considered the estimated total cost, the scale and permanency of improvements, the amount of labor required, and the frequency at which improvements occurred. To rate the intensity of *Social CPTED* activities, the research team considered the frequency and scale of activities, the degree to which they fostered community participation, and the costs and labor invested by community partners. We have listed the intensity dimensions we considered for each physical and social CPTED strategy and an explanation of how we made our ordinal intensity ratings in Table 1.

**Table 1:** CPTED strategy definitions and ordinal intensity ranking criteria.

CPTED strategies	Strategy definitions	Ordinal intensity ranking criteria (with examples)
Physical CPTED		
Territorial reinforcement	Improvements that express ownership and investment or distinguish between public and private spaces Intensity dimensions: cost, scale, permanency	Low (1): less expensive, smaller scale, or temporary installations (e.g., holiday lights, temporary art, small garden) Medium (2): moderately expensive, medium scale, or more permanent installations (e.g., landscaping, community garden) High (3): more expensive, larger scale, and permanent installations (e.g., building construction, median installations)
Surveillance	Improvements that increase visibility of streets and properties and organized surveillance by volunteers or law enforcement Intensity Dimensions: Cost, Permanency, Labor	Low (1): less expensive and less permanent improvements focused on improving visibility (e.g., shrub pruning, home light installation) Medium (2): moderately expensive and more permanent improvements focused on improving visibility (e.g., tree removal) or increasing active volunteer surveillance (e.g., Crime Watch, AmeriCorps patrols) High (3): more expensive, more permanent improvements focused on increasing visibility or involving professional surveillance by law enforcement (e.g., code enforcement, police patrols)
Access control	Improvements that guide preferred movement through spaces or block access using natural elements (e.g., landscaping), mechanical elements (e.g., locks, barriers), or active security Intensity Dimension: Cost, Scale, Labor	Low (1): less expensive, smaller scale improvements (e.g., boarded windows and doors; lock installation) to guide or block access Medium (2): moderately expensive, medium scale elements to guide or block access (e.g., fence construction) or engaging volunteer security High (3): more expensive, larger scale elements to guide or block access (e.g., security walls) or involving paid security guards
Legitimate activity support	Improvements that communicate norms for appropriate use of spaces space use and encourage positive activities Intensity Dimensions: Cost, Scale	Low (1): less expensive, smaller scale improvements focused on guiding positive community use (e.g., directive signage, installing a bench) Medium (2): moderately expensive, medium scale improvements, supporting increased community use (e.g., installing disc golf course equipment and bike share stations) High (3): more expensive, larger scale improvements focused on increasing community use (e.g., renovating a shuttered building, installing a bike lanes)
Maintenance	Removing damaged features or improving existing features to upgrade their appearance or ensure continued, intended use of the space Intensity Dimensions: cost, scale, permanency, labor	Low (1): less expensive, smaller‐scale, and less permanent repairs or improvements requiring low effort (e.g., one‐time clean‐ups on a single property, grass mowing, leaf raking) Medium (2): moderately expensive, medium scale, and more permanent repairs or improvements requiring moderate effort (e.g., extensive landscape maintenance, multi‐day mowing or gardening efforts on multiple properties) High (3): more expensive, larger scale and more permanent repairs requiring higher effort (e.g., painting multiple buildings, replacing roofs)
Social CPTED		
Social cohesion	Organized social interactions focused on strengthening relationships among residents Intensity dimensions: frequency, community participation	Low (1): one‐time events with limited social interaction opportunities and with residents from different neighborhoods (e.g., community exposition) Medium (2): one‐time events with more social interaction opportunities or mostly interactions among neighbors within the same neighborhood (e.g., neighborhood picnic, neighborhood block party) High (3): recurring events with opportunities for more social interactions (e.g., food truck nights with lawn games, conflict resolution meetings)
Community events	Activities that bring residents together with no or limited structured social interaction opportunities (e.g., concerts, sporting events, parades, education events) Intensity Dimensions: Frequency, Community Participation	Low (1): one‐time events with fewer than 30 residents Medium (2): recurring events with fewer than 30 residents or one‐time events with 30‐100 residents High (3): recurring events with 30 or more residents or one‐time events with more than 100 residents
Resident involvement	Activities where residents lead or participate in CPTED activity planning or implementation. Intensity Dimensions: Community Participation	Low (1): residents attend with limited engagement in CPTED planning or implementation (e.g., information sessions). Medium (2): residents are engaged in either planning OR implementing CPTED activities. High (3): residents are engaged in planning AND implementing CPTED activities.
Capacity building	Activities that train residents to apply CPTED strategies or build community ownership of CPTED (e.g., establishing block clubsor other neighborhood groups focused on CPTED implementation) Intensity Dimensions: Frequency Community Participation, Scale	Low (1): one‐time training events with fewer participants and focused on improving single or smaller properties. Medium (2): one‐time training events with fewer participants and focused on improving multiple properties, larger properties, or commercial businesses. High (3): multiple, recurring training events with more participants and focused on improving multiple properties, larger properties, or commercial businesses.
External supports	Activities supported with resources (e.g., funding, in‐kind labor, or materials) from external organizational partners Intensity Dimensions: Cost, Labor, Frequency	Low (1): contributed limited resources for a one‐time event. Medium (2): contributed moderate resources for a one‐time event or recurring events. High (3): contributed extensive resources for a one‐time event or recurring events.

When a CPTED activity was implemented across multiple street segments, each street segment was assigned the intensity score for that activity. We summed physical and social CPTED intensity scores per street segment per intervention year from 2015 to 2018 to create aggregate CPTED intensity scores for all street segments in the study area.

**Physical CPTED activity intensity ratings.** We assigned intensity ratings for *Territorial Reinforcement* for physical improvements to streetscapes or parcels that expressed ownership based on the cost, scale, and permanency of the improvement. The intensity ratings for *Surveillance* reflected the cost and permanency of visibility improvements and the amount of labor required (i.e., presence of organized surveillance by community residents or law enforcement). For the *Access Control* ratings, we focused on the costs and scale of improvements to guide or block movement through spaces and the amount of labor required (i.e., presence of volunteer or professional security guards). The ratings for *Legitimate Activity Support* were based on the costs and scale of improvements intended to increase desired use of the space. Finally, the *Maintenance* ratings focused on the cost, scale, and permanency of efforts to remove or improve existing features and the amount of labor required to implement these improvements. If an activity failed to meet the criteria for any of the five physical CPTED categories, it was assigned an intensity rating of zero. Detailed descriptions of the five physical CPTED categories, intensity rating criteria, and example activities are listed in Table 1.

**Social CPTED activity intensity ratings.** Intensity ratings for *Social Cohesion* focused on the frequency of activities (i.e., one‐time vs. recurring) and the degree to which these activities created opportunities for neighbors to participate and interact in ways that built bonds (as opposed to incidental social interaction). Intensity ratings were assigned for *Community Events* based on their frequency and level of community participation (i.e., attendance). For *Resident Involvement*, intensity ratings were based on the level of community participation, defined as whether residents had opportunities to merely attend an activity versus plan or implement the activity. *Capacity Building* intensity ratings focused on the level of participation (i.e., attendance), scale, and frequency of activities that built community knowledge to critically apply CPTED and expand community ownership of CPTED activities. Intensity ratings for *External Supports* were based on the amount and frequency of financial, material, and in‐kind labor resources provided by external partners to support the CPTED activities. If an activity failed to meet the criteria for any of the five social CPTED categories, the activity was assigned an intensity rating of zero. Detailed descriptions of the five Social CPTED categories, intensity rating criteria, and examples are listed in Table 1. We summed physical and social CPTED intensity scores to create aggregate CPTED intensity scores for all street segments in the UAC. The annual intensity score of CPTED activity during the intervention period (2015–2018) for the entire study area ranged from 1,511 in 2015 to 11,425 in 2017 (*M* = 7,205.0, SD = 4,171.2).

#### Violent crime incidents

Violent crime and violent firearm crime incident density values for 505 street segments in the UAC study area were derived from crime incident reports that the Flint Police Department submitted to the Michigan Incident Crime Reporting system from 2014 (1 year before the start of CPTED implementation activities) to 2019 (1 year following implementation of CPTED activities). We used two crime indices in our analyses. The first index was a density score for all Uniform Crime Report Part I violent crimes (i.e., aggravated assaults, rapes, murders, and robberies) and crimes involving firearms or other weapons, an injured victim, or domestic violence. The second index was a subset of the first index: a density score for all Part I violent crimes involving a firearm. The annual number of violent crimes during the study period (2014‐2019) in the entire study area ranged from 90 in 2018 to 146 in 2016 (*M* = 127.2, SD = 19.7). The annual number of violent firearm crimes ranged from 28 in 2018 to 74 in 2016 (*M* = 61.0, SD = 16.7). Incident crime counts were then smoothed by creating 50 m raster surfaces of kernel density estimates for each year during the 6‐year study period. The process of kernel density estimation smooths the point incident data to account for crime density in spaces near surrounding street segments. The rationale for this data‐smoothing procedure was to help address potential concerns related to the zero‐inflated nature of violent crime (i.e., relatively rare events). No street segment had zero values during any year because crime incidents occurring on nearby street segments cause non‐zero density estimates, enabling us to account for street segments in high crime areas without violent crime incidents. A similar analytic strategy was employed by Gong et al. (2023) in their assessment of Busy Streets Theory and community‐engaged greening.

#### Contextual factors

**Neighborhood disadvantage.** We calculated an index of neighborhood disadvantage at the block group level using 5‐year estimates from the American Community Survey [2015‐2019], and then assigned these values to street segments contained within the block groups (U.S Census Bureau, 2018). All street segments in the UAC area were assigned a score using this method. To compute the score, we averaged four household‐level variables related to economic and environmental neighborhood conditions: percent households in poverty, percent households on public assistance, percent renter‐occupied households, and percent vacant households (Burt et al., 2022). The statistical mean for neighborhood disadvantage was 0.31 (SD = 0.09). Higher values represented greater disadvantage.

**Property maintenance ratings.** Trained observers used the Parcel Maintenance Observation Tool (PMOT) to assess 3,140 parcels on 351 street segments in the UAC during the summer of 2016. The reliability and validity of PMOT were established in previous psychometric analyses (Reischl et al., 2016). The observers assessed the following features on any buildings: broken or boarded windows, broken doors, graffiti, fire damage, and adornments. They also rated the external surfaces of any buildings on the parcel in terms of exposure to weather (5‐point scale) and the maintenance of the surfaces (7‐point scale). We averaged standardized *z* scores for each building maintenance indicator to compute a *Building Maintenance Scale*. The observers also assessed the maintenance of gardens and shrubs (4‐point rating), the mowing/weeding of mowable areas (7‐point rating), and litter and trash (5‐point rating). We averaged standardized *z* scores for these three ratings to compute a *Lawn Maintenance Scale*. Because not every street segment in the UAC had a parcel assessed in 2016, we used inverse distance‐weighted interpolation to estimate building and lawn maintenance scores for street segments with missing PMOT data (*n* = 106). The mean score for building maintenance was −0.01 (SD = 0.45). The mean score for lawn maintenance was 0.08 (SD = 0.48). Higher scores for both scales indicated better property maintenance.

### Analytic approach

**Figure 1:**
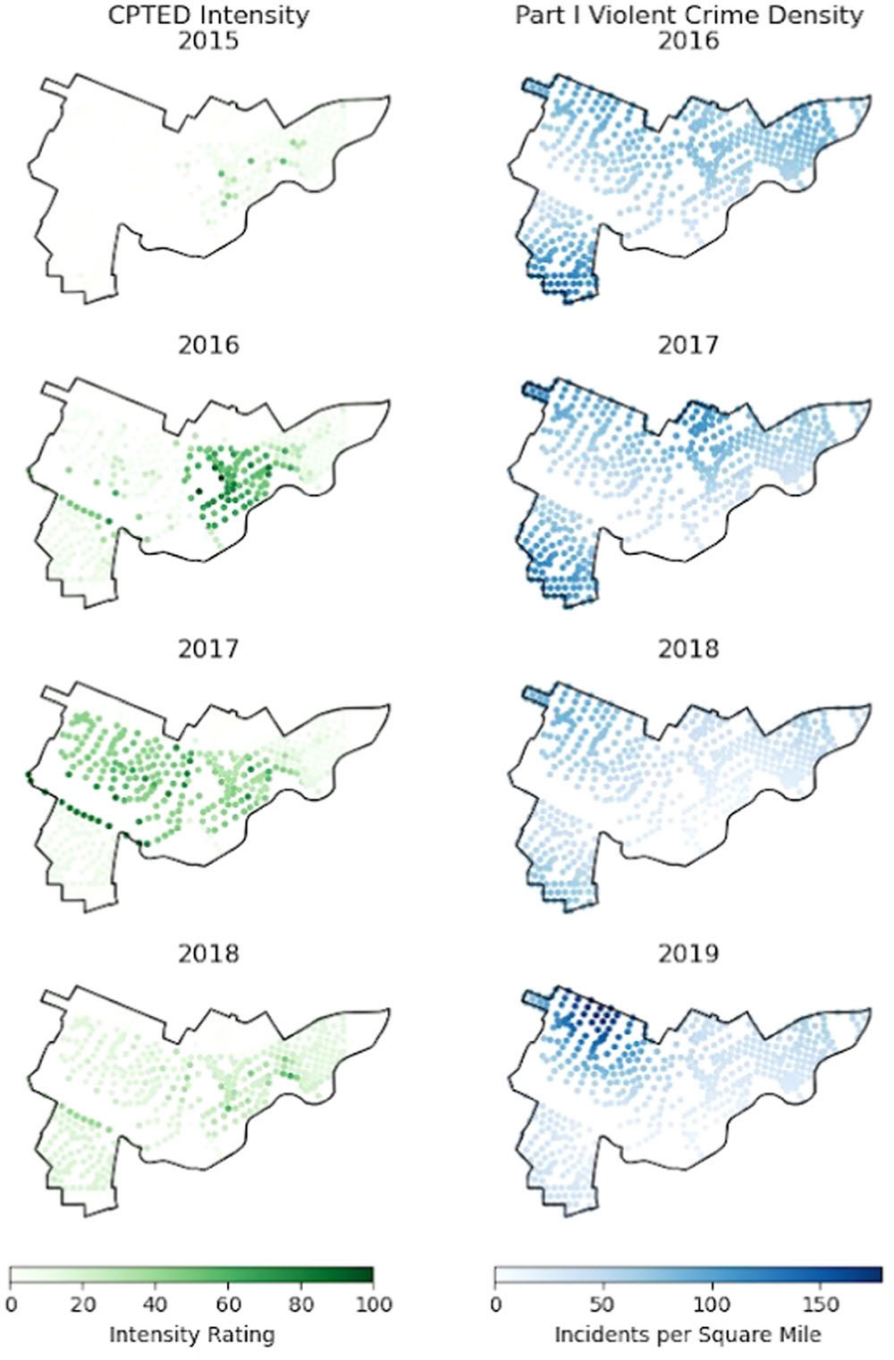
CPTED intensity by intervention year (left) and Part I violent crime density for subsequent year (right).

Measures of central tendency (e.g., mean, median) and dispersion were examined to characterize CPTED intensity (i.e., the aggregate of physical and social CPTED intensity), Part I violent crime and violent firearm crime densities, and all other predictors across 505 street segments. Due to collinearity concerns between physical and social CPTED intensity (*r* = .86, *p* < .001; VIF > 3; Johnston et al., 2018), we summed the physical and social CPTED intensity scores, generating aggregate CPTED intensity scores for each street segment in the UAC for each year of the intervention period, 2015–2018 (See Figure 1). For each annual estimate of CPTED intensity, the violence densities in the following year were the outcomes (e.g., CPTED intensity in 2015 predicting violent crime density in 2016) (See Figure 1). We employed linear mixed effects regression models with random intercepts for the study area street segments to estimate whether CPTED intensity contributed to reductions in A) density of Part I violent crime, and B) density of Part I violent crime incidents involving a firearm over time. Regression analyses were an iterative process, beginning with a baseline model including only an intercept, year, baseline Part I violent crime density (average of 2014 and 2015 densities), and disadvantage index as predictors (Model I). Next, property maintenance variables (i.e., building and lawn maintenance) were added (Model II). An interaction term for CPTED intensity and year was then added to the regression (Model III). A final specification was explored, which included a spatial lag for violent crime density (Model IV). Inclusion of a spatially lagged term was based on the finding of global spatial autocorrelation in the Model III residuals for violent crime density (Moran's *I* = 0.28, *p* < .001) and violent firearm crime density (Moran's *I* = 0.35, *p* < .001). Akaike information criterion (AIC) and Bayesian information criterion (BIC) estimates were produced for each model to assess fit, and *X* ^*2*^ tests were conducted to estimate model fit of the final model (Model IV) compared to the reduced aspatial model (Model III). All models were estimated using R (R Core Team, 2021).

## RESULTS

**Figure 2:**
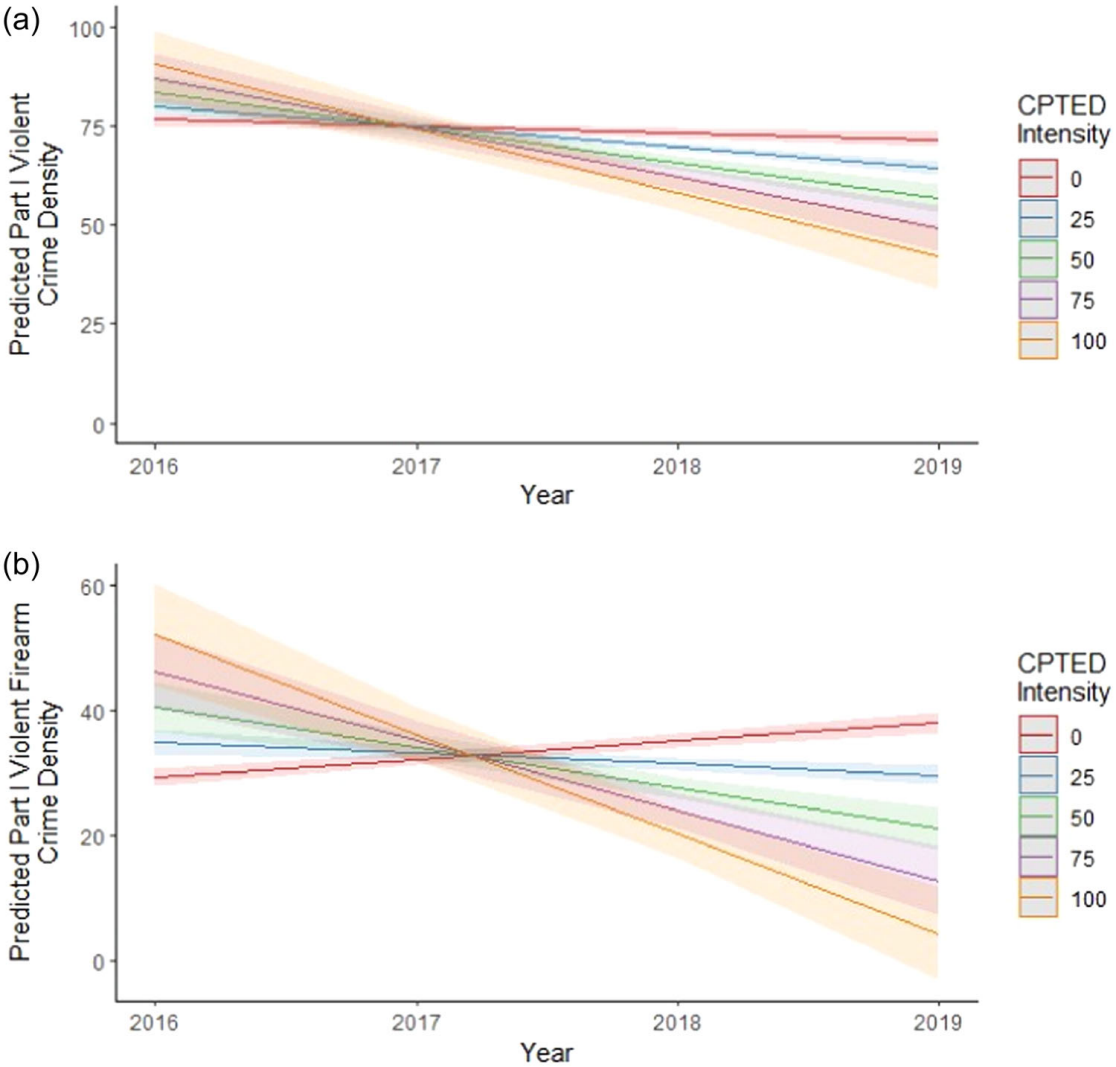
Interaction effects between CPTED intensity by year and the association with part I violent crime density (a) and part I violent firearm crime density (b).

A summary of the mixed effects regression analyses is reported in Table 2. In the full model specification (Model IV), after adjusting for baseline violent and violent firearm crime, neighborhood disadvantage, property maintenance, the interaction between community‐engaged CPTED intensity and year, and spatial autocorrelation, we observed that higher levels of community‐engaged CPTED intensity were associated with steeper declines in violent crime density over time (*β* = −0.14, *p* < 0.001). Similarly, higher levels of community‐engaged CPTED intensity were associated with steeper declines in violent firearm crime density over time (*β* = −0.19, *p* < 0.001). To illustrate the significant interaction effects between year and community‐engaged CPTED intensity, we conducted post hoc analyses that provided simple slopes for our full model specification (Model IV). Figure 2 presents a visualization of the relationship between year and Part I violent crime density (A), and between year and Part I violent firearm crime density (B), when community‐engaged CPTED intensity is held at five unique values. For both violence outcomes, higher levels of CPTED intensity were associated with steeper declines in violence density over time. Compared to the reduced model (Model III), which excluded spatially lagged crime density, the full model specification (Model IV) explained significantly more variation in the Part I violent crime density trend (*X* ^*2*^ = 659.66, *p* < 0.001). The full model also explained more variation in the Part I violent firearm crime density trend compared to the reduced model (*X* ^*2*^ = 377.83, *p* < 0.001). These findings were further supported by lower AIC and BIC values in the full model compared to the aspatial models (Models I‐III; Table 2). Testing for spatial autocorrelation in the model residuals with the inclusion of spatially lagged violent crime density revealed reduced spatial autocorrelation compared to Model III, though still statistically significant (violent crime density: Moran's *I* = 0.24, *p* < 0.001; violent firearm crime density: Moran's *I* = 0.31, *p* < 0.001).

**Table 2:** Mixed effects regression model summary.

	Model I		Model II		Model III		Model IV	
	Violent crime	Violent firearm crime	Violent crime	Violent firearm crime	Violent crime	Violent firearm crime	Violent crime	Violent firearm crime
**Predictor**	*Est*.	*Est*.	*Est*.	*Est*.	*Est*.	*Est*.	*Est*.	*Est*.
Intercept	−6.22**	−4.17*	−4.36	−3.90	0.19	−1.48	−31.68***	−33.31***
Year	−7.68***	−3.04***	−7.68***	−3.04***	−5.13***	−0.13	−1.67***	2.95***
Baseline violence density	1.28***	1.12***	1.27***	1.12***	1.21***	1.07***	1.10***	0.96***
Disadvantage index	−13.12*	9.39	−13.78**	8.86	−18.91***	1.32	−36.32***	−19.34***
Building maintenance	–	–	−4.01***	−2.23*	−3.85***	−1.50	−3.09***	0.06
Lawn maintenance	–	–	−4.30***	−2.37**	−3.88***	−2.13*	−2.80***	−1.45
CPTED intensity	–	–	–	–	0.17**	0.26***	0.14**	0.23***
CPTED intensity*Year	–	–	–	–	−0.20***	−0.25***	−0.14***	−0.19***
Spatial lag violence density	–	–	–	–	–	–	0.53***	0.49***
AIC	17593	17300	17534	17280	17471	17190	17098	16821
BIC	17627	17333	17579	17324	17527	17246	17159	16883

*Note*: Model I includes year, baseline violence density, and disadvantage index as predictors; Model II includes Model I predictors + property maintenance predictors; Model III includes Model II predictors + CPTED intensity*year interaction; Model IV includes Model III predictors + violence density spatial lag.

Abbreviations: AIC = Akaike information criterion, BIC = Bayesian information criterion, CPTED = Crime prevention through environmental design, Est = Estimate.

*** *p* < 0.001

** *p* < 0.01

* *p* < 0.05.

## DISCUSSION

We found that the intensity of community‐engaged CPTED was associated with declines in violent and firearm crime. Our study suggests that CPTED implemented by a community coalition is a promising intervention to curb serious violent crime, including firearm violence. This is consequential as we have few evidence‐based, community‐driven interventions for violence prevention that are focused on addressing neighborhood‐level risks versus promoting individual‐level behavior change (Branas et al., 2017; Cunningham et al., 2019; Ngo et al., 2019). This attention to neighborhood change is urgently needed as interpersonal violence and firearm violence are public health crises that disproportionately affect disinvested cities with high rates of vacancy and physical disorder (Branas et al., 2013; Goldstick et al., 2022). Addressing deteriorated neighborhood conditions to reduce violence may be key to ameliorating the wide‐sweeping, adverse outcomes associated with violence exposure, including poor mental health (Abba‐Aji et al., 2024), chronic disease (Hsieh et al., 2017), criminal justice involvement (Carter et al., 2018; McGee et al., 2017) and cyclical violence (Rowhani‐Rahbar et al., 2015).

Our study builds upon a growing body of research indicating that community‐engaged interventions to improve neighborhood built environments can support reductions in violent crime (Bushman et al., 2023; Gong et al., 2023; Heinze et al., 2018; Kondo et al., 2016; Pizarro et al., 2020) and firearm violence (Bushman et al., 2023). To date, researchers have primarily found violence prevention effects for built environment interventions focused on maintaining vacant properties (i.e., mowing, trash removal) (Bushman et al., 2023; Heinze et al., 2018; Pizarro et al., 2020) or repurposing vacant lots for community needs like gardens or play spaces (Gong et al., 2023; Kondo et al., 2016). Our study expands this evidence base by evaluating coalition‐led CPTED projects incorporating physical and social strategies. This is notable, as the systematic application of physical and social CPTED has not been rigorously evaluated for community‐level violence prevention. In one prior study, Lee et al. (2023) inventoried neighborhood features related to physical CPTED principles and found that features related to maintenance (i.e., street cleanliness, visual quality of buildings) were negatively associated with violent crime in neighborhoods surrounding economically vulnerable schools. Yet this study was cross‐sectional and did not include an assessment of social CPTED principles or CPTED intensity. Our study adds to these findings by examining the effects of both physical and social CPTED intensity on community‐level violence across time. To our knowledge, our study is also the first to explicitly examine the intensity of physical and social CPTED for reducing firearm crime.

### Implications for theory

Our results support and inform the development of Busy Streets Theory (BST; Aiyer et al., 2015). BST suggests that active resident participation in improving vacant and deteriorated environments can promote safer communities by reducing opportunities for crime and asserting positive community ownership and oversight (Aiyer et al., 2015; Reynald, 2011; Wilcox & Cullen, 2018). BST further posits that residents who collaborate to improve neighborhood environments, build social capital and social control that enhance their capacity to monitor and prevent neighborhood crime (Aiyer et al., 2015). In a prior qualitative study of the UACC, residents reported that planning and implementing CPTED improvements supported them to build bonds and increased their social capital and efficacy to organize further neighborhood improvements (Rupp et al., 2020). Our study provides additional quantitative evidence that these community‐engaged CPTED improvements can be effective for reducing violent and firearm crime.

This study also informs the conceptualization of CPTED by developing a multi‐dimensional measure of CPTED intensity that includes both physical and social components (Cozens & Love, 2015; Saville & Cleveland, 2013). Prior evaluations of CPTED interventions have been hindered by the use of multiple frameworks with differing principles and limited focus on the intensity of interventions (Ekblom, 2011; Lee et al., 2023). Similarly, the community engagement dimensions of CPTED (i.e., Social CPTED) have been proposed as important to crime prevention (Cozens & Love, 2015; Saville & Cleveland, 2013), but have never been empirically assessed. Our study addressed these gaps, by conceptualizing ordinal intensity measures for each physical and social strategy in alignment with leading CPTED theoretical frameworks (Cozens & Love, 2015; Saville & Cleveland, 2013). We also added *Capacity Building* as a new social CPTED strategy in our intensity measure to be responsive to the forms of community engagement that we observed in the UACC's implementation. This strategy focuses on increasing community readiness to apply CPTED and is particularly relevant for evaluating Busy Streets Theory, given its focus on local control of neighborhood change. Our intensity measure may be useful for future research examining what CPTED strategies are most potent for neighborhood violence prevention.

### Implications for practice

Our results have several implications for practice and policy. First, our findings suggest that implementing more intensive, community‐driven CPTED activities can help to reduce crime in economically distressed neighborhoods. This suggests that CPTED interventions may benefit from engaging a range of collaborators (e.g., homeowners, school systems, health care systems, businesses) and implementing multiple CPTED projects to accumulate greater CPTED intensity, and increase the probability of reducing violence. Similarly, CPTED organizers might consider the range of ways that community engagement in CPTED projects can be expanded, for example, by involving residents in planning and implementing projects through participatory design processes like placemaking (Project for Public Spaces, 2022) or creating opportunities for sustained community engagement once projects are completed (e.g., replanting gardens, planning community celebrations in a refurbished park).

Second, forming community coalitions or developing the capacity of existing coalitions to lead community‐engaged CPTED efforts may be beneficial for implementing more intensive CPTED projects. In this study, the UACC mobilized support from local universities, health systems, non‐profits, and police to fund more extensive projects (e.g., demolitions, directed patrols), while also backing smaller‐scale, neighborhood‐led projects and events. In a qualitative study of the UACC, residents reported that local institutional support was instrumental for securing resources and organizing residents to create a safe foundation for more intensive, community‐engaged CPTED (Rupp et al., 2020). Coalitions promote resource‐sharing across institutions that can reduce the costs and burden of improving neighborhood conditions (Lardier et al., 2019). This is particularly important for funding and coordinating CPTED activities in communities that have diminished resources due to decades of disinvestment and discriminatory policies and practices (e.g., plant closures, white flight, racial segregation, discriminatory lending) (Dewar & Thomas, 2013; Knopov et al., 2019; Lardier et al., 2019; Sadler & Lafreniere, 2017).

Third, our results support the notion that strategies to encourage community involvement in all phases of CPTED projects are critical for success. Researchers and practitioners have consistently reported that community‐engaged improvements to the built environment are more likely to be responsive to local needs and accepted by neighborhood residents (Campbell‐Arvai & Lindquist, 2021; O'Keefe et al., 2021; Rupp et al., 2022). CPTED projects that solicit and apply resident input can help to ensure residents benefit from improvements while avoiding green gentrification that risks displacing residents and reshaping neighborhoods based on external interests (Anderson & Minor, 2017; Campbell‐Arvai & Lindquist, 2021; Rupp et al., 2020). Community involvement in planning and implementing CPTED may also enhance residents' skills, capacity, and local control of CPTED improvements (Lowe & Thaden, 2016), which can support sustainability (Faga, 2006; Lowe & Thaden, 2016; O'Keefe et al., 2021; Rupp et al., 2022; Schilling & Logan, 2008).

Finally, this study suggests the value of public and social policies that promote community‐engaged CPTED interventions as a potential alternative to costly, individual‐level criminal justice strategies (Branas et al., 2016; Frieden, 2010; Institute of Medicine et al., 2003; Rose & Clear, 1998). In 2020, state and local governments in the U.S. spent $266 billion on policing, courts, and corrections combined (Urban Institute, 2024). Criminal justice system contacts can destabilize communities socially and economically because individuals may become stigmatized, families disrupted, and communities over policed (Maroto & Sykes, 2020; Rose & Clear, 1998). Community‐engaged CPTED may be one way to reduce violence while averting some of these costs and harms. Yet notably, CPTED and policing are not mutually exclusive and can be complementary, as occurred in the UACC's implementation, in which police collaborated with community organizations and neighborhood residents to plan and implement CPTED projects (Rupp et al., 2020). These efforts align with a community policing approach which is focused on increasing trust and fostering collaborative problem‐solving to improve effectiveness while reducing harms (Department of Justice, 2014).

### Limitations

Despite our promising findings, this study had some methodological limitations. First, the CPTED activities were guided by community‐driven goals and directed by a community coalition, which prohibited experimental comparisons of similar street segments with and without CPTED activities. To compensate for this limitation, we statistically controlled for community‐level factors that affect vulnerability to neighborhood crime (Hedayati Marzbali et al., 2012; Sampson et al., 1997, 1999), including property conditions and a measure of neighborhood disadvantage that accounted for vacancy, residential tenure, and economic resources (Burt et al., 2022). Yet, we were unable to statistically control for all community‐level factors that may have influenced violent crime, such as levels of law enforcement patrols. For example, the City of Flint Police Department had limited capacity for patrols during the study period and was unable to provide detailed patrolling records. For future studies, it would be useful to obtain such records to control for law enforcement trends in predictive models.

Second, the highly community‐engaged nature of the CPTED strategies implemented across the UAC made it challenging to isolate the unique contributions of physical and social CPTED, and our physical and social CPTED measures were highly correlated as a result. Future studies that compare community‐engaged CPTED interventions with those implemented in a more top‐down fashion by institutions (e.g., police departments) with less community participation would help to distinguish the effects of physical and social CPTED strategies on violent crime. Third, we were unable to compare if certain principles were more effective in different socioeconomic contexts due to a lack of statistical power (i.e., relatively small number of street segments in our study area) and the fact that the UAC was a largely homogenous, low‐income, and residential area. Future studies that are sufficiently powered to determine what strategy combinations are more effective across a range of contexts would be worthwhile.

Finally, despite including spatially lagged violent crime density in our full model specification, we continued to observe spatial autocorrelation in model residuals, though at a reduced magnitude compared to aspatial models. This finding was not surprising, given the concentrated nature of our study area and the CPTED activities therein. Due to the clustering pattern of violent crime observed in many cities, we expect that some degree of spatial autocorrelation is inevitable, and we can only minimize it.

## CONCLUSION

These limitations notwithstanding, we found that community‐engaged neighborhood change, incorporating both physical and social strategies, was associated with reductions in violence. We found these effects for both violent and firearm crime over time after controlling for possible alternative explanations and accounting for spatial autocorrelation. These results both contribute to the development of Busy Streets Theory and provide guidance to practitioners interested in community‐engaged violence prevention that is not focused on costly and often ineffective individual‐level behavior change. Community‐engaged CPTED may be particularly beneficial because it takes a nonpunitive, population‐level approach that avoids some of the costs and harms associated with justice‐system interventions. Support from a community coalition may aid in implementing community‐engaged CPTED interventions that are more intense and sustainable, which may be more beneficial for achieving reductions in violent and firearm crime over time. The prevention of even one violent crime may avert an array of individual and societal costs (Branas et al., 2016; Corso et al., 2006; Washington State Institute for Public Policy, 2019), including physical injury, loss of productivity across the lifespan, and community‐level violence exposure (American Public Health Association, 2018; Carter et al., 2018; Rowhani‐Rahbar et al., 2015). Our results support the conclusion that collaborating with community institutions and residents on neighborhood change is a promising approach to creating busy streets and reducing violence and firearm violence in disinvested cities.

## CONFLICT OF INTEREST STATEMENT

The authors declare no conflicts of interest.
